# Methyl jasmonate induction of tanshinone biosynthesis in *Salvia miltiorrhiza* hairy roots is mediated by JASMONATE ZIM-DOMAIN repressor proteins

**DOI:** 10.1038/srep20919

**Published:** 2016-02-15

**Authors:** Min Shi, Wei Zhou, Jianlin Zhang, Shengxiong Huang, Huizhong Wang, Guoyin Kai

**Affiliations:** 1 Zhejiang Provincial Key Laboratory for Genetic Improvement and Quality Control of Medicinal Plants, Hangzhou Normal University, Hangzhou 310018, People’s Republic of China; 2 Institute of Plant Biotechnology, Development Center of Plant Germplasm Resources, College of Life and Environment Sciences, Shanghai Normal University, Shanghai 200234, People’s Republic of China; 3 Kunming Institute of Botany, Chinese Academy of Sciences, Kunming 650201, People’s Republic of China

## Abstract

Jasmonic acid (JA) is an important plant hormone involved in regulation of many aspects of plant growth and development including secondary metabolism and JASMONATE ZIM-DOMAIN (JAZ) proteins are key components in JA signal processes. In this study, two new JAZ genes named *SmJAZ3* and *SmJAZ9* were cloned from *S. miltiorrhiza* hairy roots and characterized. Expression profiles under methyl jasmonate (MJ) treatment revealed that *SmJAZ3* and *SmJAZ9* were both MJ-responsive. Subcellular localization assay showed that *SmJAZ3* was located in nucleus while *SmJAZ9* was preferentially in nucleus. Expression of *SmJAZ3* and *SmJAZ9* in *S. miltiorrhiza* hairy roots differently affected the production of tanshinone. Over-expression of *SmJAZ3* or *SmJAZ9* in hairy roots produced lower level of tanshinone compared with the control, tanshinone production was as low as 0.077 mg/g DW in line *SmJAZ3-3* and 0.266 mg/g DW in line *SmJAZ9-22*. Whereas, down-regulation of *SmJAZs* enhanced tanshione production, the content of tanshinone increased to 2.48 fold in *anti*-*SmJAZ3-3* line, and 1.35-fold in *anti-SmJAZ9-23* line. Our work indicated that *SmJAZ3* and *SmJAZ9* are involved in regulation of tanshinone biosynthesis and act as repressive transcriptional regulators in the JA signaling pathway, which paves the way to further dissect molecular mechanism in details in the future.

During the entire life of the plants, they are frequently exposed to various abiotic or biotic stresses such as high light, drought, salinity, and attack from herbivores or pathogens^1^,^2^,^3^,^4^. Overall survival of plants depends on their abilities to quickly perceive and respond to external challenges, which is regulated by several signaling pathways such as jasmonic acid (JA) signaling. JA signaling has been investigated for several decades, and is probably the best-characterized oxylipins. The signaling pathways modulate plant defense responses against biotic and abiotic challenges combining a complex network^4^,^5^,^6^,^7^. Methyl jasmonate (MeJA), a fragrance from the jasmine flower, has been used as a common ingredient in perfumes for a long time. MeJA and its related members (referred as JAs) are fatty acid-derived oxylipins regulating many aspects of plant growth, development and defense^6^,^8^. They are widely regarded as regulators in defense to environmental stresses such as pathogen and pest attack, wounding, ozone exposure, ultraviolet radiation and salt stress^9^,^10^,^11^. Also they participate in controlling plant development^5^,^12^,^13^. JAs derive from α-linolenic acid in the chloroplast, through a series of reactions such as cyclation and reduction, to generate unstable (+)-7-iso-JA, form more stable JA^14^,^15^,^16^. After conjugation with amino acids by Jasmonate Resistant1 (JAR1), JAs can be synthesized into a variety of products including the methyl ester (JA-Me), and (3 R,7 S)-jasmonoy l-L-isoleucine or JA-Ile which was recently described as the molecularly active form of the hormone. JA-Ile associates with an F-box protein COI1, forms the E3-ubiquitin ligase complex and leads to the degradation of JASMONATE ZIM-DOMAIN (JAZ) repressors by the 26 S proteasome^17^. Degradation of JAZ repressors releases MYC2 transcription factor(s) through repression or activation the transcription of genes involved in plant defense responses^15^,^18^. The presence of bioactive JA-Ile, therefore, directly determines the duration of plant defense responses to biotic stresses that elicit JA signaling pathway.

JAZ family, interact with MYC2 transcription factor and repress its function in JA signaling, and act as repressors^17^,^19^. Twelve JAZ proteins are found in *Arabidopsis*, they share a conserved TIFY×G motif within the ZIM (or TIFY) domain in the N-terminus and a SLX2FX2KRX2RX5PY consensus motif within the Jas domain in the C-terminus. ZIM domains mediate the homo- and heteromeric interactions among the JAZ proteins as well as their interaction with other transcription factors^20^,^21^,^22^. In the presence of JA-Ile, JAZ proteins are recognized by Skp1-Cul1-F-box protein (SCF)-type E3-ubiquitin ligase complex, consequently got degraded by the 26 S proteasome. On the contrary, JAZ repressors bind to transcription factors (TFs) by recruiting the general co-repressors TOPLESS (TPL) and TOPLESS-Related (TPR) proteins through an interaction with the adaptor protein NINJA to prevent their activity. Up to now, JAZs have been testified to interact with some other TFs to mediate many process of development including trichome development and flavonoids synthesis.

*Salvia miltiorrhiza* Bunge, a well-known traditional Chinese herb, plays a crucial role in clinical treatment of cardiovascular and cerebrovascular diseases^23^,^24^. There are two kinds of active components in *S. miltiorrhiza*, one kind is the liposoluble tanshinone containing cryptotanshinone, tanshinone I, tanshinone IIA, dihydrotanshinone etc., which share a variety of biological activities including anti-oxidant, heart-protective, anti-ischemic, anti-bacteria and anti-tumor properties^23^,^25^,^26^,^27^,^28^. The other kind is hydrosoluble phenolic acids including caffeic acid, lithospermic acid A, B, C and rosmarinic acid^24^,^26^,^27^. Diterpene tanshinone is mainly synthesized via the methylerythritol phosphate (MEP) pathway, with some degree of crosstalk between the MEP pathway and the mevalonate (MVA) pathway^23^. Enhanced accumulation of tanshinone elicited by addition of exogenous JA treatment in *S. miltiorrhiza* has been reported^28^,^29^, but the exact molecular regulation mechanism are still unclear. Recently, several tanshinone biosynthetic genes such as *SmHMGR*, *SmDXR*, *SmHMGS*, *SmGGPPS* and *SmCYP76AH1* have been successfully cloned from *S. miltiorrhiza*^25^,^27^,^30^,^31^,^32^, which provided the foundation to understand molecular mechanism of tanshinone biosynthesis induced by MJ in *S. miltiorrhiza*. As a key regulator in plant JA signal pathway reported recently, JAZ repression proteins should have important regulatory role in tanshinone biosynthesis in *S. miltiorrhiza*. However, little is known about exact role of JAZ on tanshinone biosynthesis in *S. miltiorrhiza*. Herein, two genes of JAZ family were isolated from *S. miltiorrhiza* and their roles in regulating the tanshinone biosynthetic pathway are functionally identified through genetic engineering strategy.

## Materials and Methods

### Experimental materials

*S. miltiorrhiza* plants were grown in pots in the greenhouse under 25 °C with 16 h light/8 h dark period and relative air humidity of 60% or cultured on MS medium (pH5.8) containing 3% sugar and 0.8% agar as reported before^23^,^24^,^29^. Two-year-old flowering *S. miltiorrhiza* plants were obtained from the Plant Garden of Shanghai Normal University and used for the analysis of expression pattern of *SmJAZs* in taproot, fibril, leaf, petiole, stem, seed and flower. The seeds of *N. benthamiana*, plasmid *pCAMBIA2300*, *pGBKT7* and *pGADT7* were provided by Professor Kexuan Tang from Shanghai Jiaotong University. *Escherichia coli* DH5α, *Agrobacterium* C58C1 and GV3101 were kept in our own laboratory. The plasmid of *pMON530* was gifted by Professor Zhongnan Yang from Shanghai Normal University. The pMD-18 T vector and reverse-transcription PCR Kit were purchased from TaKaRa Biotechnology Co., Ltd. Primers synthesis and DNA sequencing was performed by Shanghai Sangon Biotechnological Company, China. Standards of cryptotanshinone, tanshinone I, tanshinone IIA, dihydrotanshinone were purchased from Aladdin, China. All other chemicals used were of analytical grade.

### Isolation of two JAZs from *S. miltiorrhiza*

The hairy roots of *S. miltiorrhiza* were dried and pulverized in liquid nitrogen with mortar and pestle for extraction of the total RNA. Total RNA was used to synthesize the first strand cDNA with primer AP (5′-GGCCACGCGTCGACTAGTAC(T)_16_-3′) by using RT-PCR system (TaKaRa, Japan). A local *S. miltiorrhiza* transcription database was built up from public transcripts from NCBI as well as our own transcriptomic data sequenced by hairy roots. Conserved domains of ZIM and Jas were utilized to search our transcriptome database to identify putative JAZs from *S. miltiorrhiza* with Blastx and Blastn. ORF Finder was used to identify the length of the ORF. Among the candidate JAZ sequences, *SmJAZ3* (lack of 3-terminal) was obtained with the specific forward primer *SmJAZ3-F421* (5′-GCCGTTGGAACCACTGATTTTAG-3′) together with the primer AUAP. Primer pairs (*SmJAZ9-F*: 5′-ATGGAGAGAGATTTCATGGGGT-3′, *SmJAZ9-R*: 5′-TCAGTCATCCTTGCTGACGGA-3′) were synthesized to amplify the whole ORF of *SmJAZ9*. The PCR amplification conditions were as follows: initial denaturation at 95 °C for 8 min, followed by 35 cycles denaturation at 95 °C for 30 s, annealing at 58 °C for 1 min and extension at 72 °C for 1 min 30 s.

### Bioinformatics analysis of *SmJAZs*

Several bioinformatics softwares and websites were used for analysis of the two *SmJAZs*. The nucleotide sequence and open reading frame (ORF) were analyzed by ORF Finder and the sequence comparison was conducted through database search using BLAST (NCBI, http://www.ncbi.nlm.nih.gov). Alignment of nucleotide and deduced amino acid sequences containing the two *SmJAZs* and other JAZs such as *AtJAZs* and *OsJAZs* were aligned with the ClustalW with default parameters. MEGA5.1 combined with CLUSTAL W alignments were used to construct the phylogenetic tree.

### Expression profiles of *SmJAZ* genes

To investigate the expression pattern of the *SmJAZs* in different tissues (taproot, fibril, leaf, petiole, stem, seed and flower) as well as its expression at 0 h, 0.5 h, 1.0 h, 2.0 h and 4.0 h after 100 μM MeJA treatment, plant materials were collected accordingly and total RNA was isolated according to the method described before^23^,^24^. Reverse transcription (RT) reaction was carried out using the kit (TaKaRa, Japan) with 20 μL volume consisting of 4 μL 5×M-MLV buffer, 2 μL 50 μM primer AP, 1 μL 10×dNTPs, 0.5 μL 200 U/μL RNase M-MLV and 0.5 μL 40 U/μL RNase inhibitor at 42 °C for 1.5 h, then inactivated at 70 °C for 15 min^24^. The qRT-PCR reaction was carried out using the Super Real PreMix kit (Tiangen, China) and performed on the Applied Biosystem StepOne Real Time PCR System (Applied Biosystems, USA) with an optional 48-well plate. The house-keeping gene (*SmActinF*: 5′- AGCACCGAGCAGCATGAAGATT-3′; *SmActinR*: 5′- AGCAAAGCAGCGAACGAAGAGT-3′) was performed as an internal control to estimate the expression level of the *SmJAZs* according to the relative quantitative analysis method (2^−∆∆CT^). Amplifications were performed under the following condition: 10 min denature at 95 °C, then 40 cycles of 15 s denature at 95 °C, 30 s annealing at 60 °C and 30 s extensions at 72 °C^24^,^33^.

### Subcellular localization of *SmJAZs*

*Bgl*II and *Kpn*I site were added to the 5′- and 3′- of the ORF of *SmJAZ3* and *SmJAZ9* respectively via PCR. The PCR products were digested with *Bgl*II and *Kpn*I and cloned into the vector *pMON530*. The constructed vectors were transferred into *Agrobacterium* strain ASE. Forty-day-old tobacco plants were used for the transient transformation experiment. After two days′ injection, localization of *SmJAZ*s was observed using the confocal microscope (Carl Zeiss).

### Construction of plant expression vectors and hairy root cultivation

The *SmJAZ3/SmJAZ9* gene or *anti-SmJAZ3/anti-SmJAZ9* was amplified via PCR as indicated above. The vectors *pCAMBIA1304* and *pCAMBIA2300* were double-digested with *Hin*d III and *Eco*R I. The smaller DNA fragment containing GUS gene expression cassette from *pCAMBIA1304* was ligated with the large *pCAMBIA2300* fragment to obtain the modified vector *pCAMBIA2300*^+^. The complete ORF of *SmJAZ*s with restriction sites *Spe*I and *Bst*EII were firstly cloned into *pMD-18 T* to generate *pMD18T-SmJAZ*s. *pCAMBIA2300*^+^ and *pMD18T-SmJAZ*s were then double-digested with *Spe*I and *Bst*EII. The complete ORF of *SmJAZ*s were then cloned into *pCAMBIA2300*^+^ to replace the GUS gene of the GUS gene expression cassette from *pCAMBIA1304*, under the control of CaMV35S promoter and the NOS terminator to generate *pCAMBIA2300*^+^-*SmJAZ3* and *pCAMBIA2300*^+^-*SmJAZ9*. *pCAMBIA2300*^+^-*anti-SmJAZ3* and *pCAMBIA2300*^+^- *anti-SmJAZ9* were constructed using similar strategy with opposite direction of ORF in place of GUS gene. These above constructed plastids were transformed into *A. rhizogenes* strain C58C1 respectively. The aseptic leaves of 30 day-old *S. miltiorrhiza* were cultured in 1/2 MS medium in darkness for two days and then immersed in the *Agrobacterium* suspension for 10 min, then co-cultivated on MS solid medium^23^,^29^. After 2 days, the leaves were transferred to 1/2 MS containing carbencillin (300 mg/L) and subcultured to 1/2 MS with reducing concentration of cefotaxime (300 mg/L, 100 mg/L) every two weeks. After 6 to 7 weeks, about 3-4 cm long hairy roots were excised from the leaves. The empty *pCAMBIA2300* vector was used as a vector-only control. DNA isolation and PCR analysis procedure were carried out as we reported before^23^,^24^,^28^ Positive colonies were cut down and moved to 100 mL 1/2 MS medium in 250 mL Erlenmeyer flasks shaking with the speed of 100 rpm in darkness at 25 °C and harvested after 60 days for RNA and tanshinone extraction.

### Tanshinone extraction and HPLC analysis

After 2 month culture, the hairy roots were dried at 50 °C in an oven and then ground into powder, 100 mg powder was placed in 16 mL solvent of methanol/dichloromethane (3:1, v/v) for tanshinone extraction, and then kept in dark for 24 hours after 1 hour′s ultrasonic process with frequency of 40000 Hz at room temperature (Kunshan HC-2002 S, China). The substance was dried under vacuum and re-dissolved in 2 ml methanol. The solution was filtered and used for HPLC on Agilent 1260 apparatus equipped with a Waters reversed-phase C18 symmetry column. Acetonitrile-water (65:35, v/v) worked as the mobile phase at a flow rate of 1 mL/min with the detection wavelength at 270 nm^5^,^7^. Chromatographic peaks were integrated and compared with the respective standard curves to calculate the individual and total amount of tanshinone^23^,^24^. The sum of HT, CT, T1 and T2A were calculated as total tanshinone (TT) in this paper.

### Yeast two-hybrid assays

To assess possible protein interactions, the corresponding plasmids (*pGBKT7*-*SmJAZs* and *pGADT7*-*AtMYC2*) were co-transformed into *Saccharomyces cerevisiae AH109* cells following standard protocols^5^,^19^. Both the *SmJAZ3* and *SmJAZ9* cDNAs were cloned into *pGBKT7* vector and the cDNA of *AtMYC2* was inserted into *pGADT7*. Plasmids *pGBKT7*-*AtJAZ9* and *pGADT7*-*AtMYC2* were co-transformed into *AH109* as the positive control. The empty vector *pGADT7* and *pGBKT7* constructs were co-transformed as the negative control. The indicated construct pairs were transformed into yeast *AH109* and screened on yeast synthetic drop-out-Trp-Leu at 30 °C. Three days after transformation, yeast colonies were grown in selective liquid media for about 7 h, and the cell density was adjusted to OD_600_ = 1. A volume of 10 μL sample of the cell suspensions was plated out on yeast synthetic drop-out (SD/-Trp-Leu-His-Ade) to test protein interaction. Plates were incubated at 30 °C for 2–4 days.

### Statistical analysis

All the experiments including culture of hairy root clones, PCR identification, qRT-PCR and HPLC analysis were repeated three times. Results of tanshinone content were presented as mean values ± SD. The error bars were from biological triplicates. The statistical significant difference was analyzed by one sample T test, and the difference of various hairy root lines were used in the one-way analysis of variance (ANOVA) using SPSS 11.5 software (SPSS, Inc.).

## Results

### Isolation and bioinformatics analysis of *JAZ*s from *S. miltiorrhiza*

**Figure 1:**
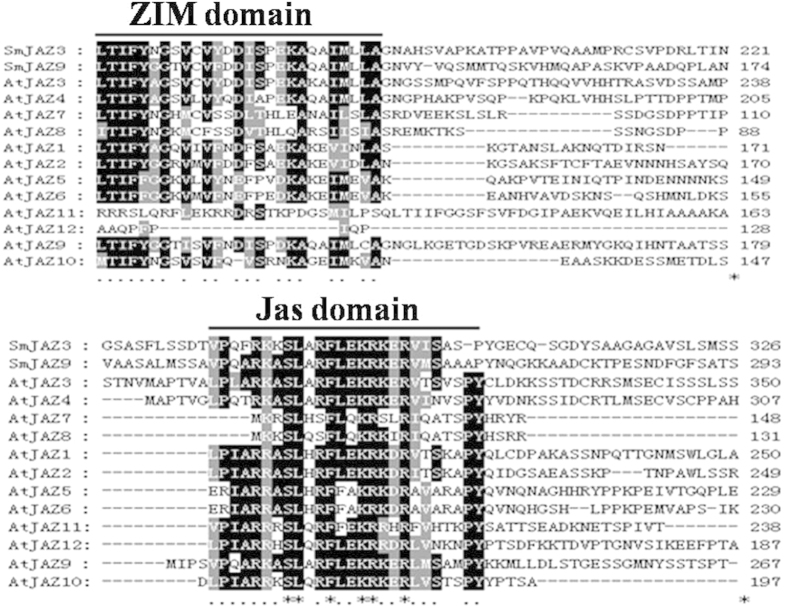
Multiple alignments of SmJAZs with JAZs from *Arabidopsis thaliana.* AtJAZ1 (NP973862.1), AtJAZ2 (AAP13409.1), AtJAZ3 (NP001078174.1), AtJAZ4 (AAX55088.1),AtJAZ5 (AAO00903.1), AtJAZ6 (AAL15195.1), AtJAZ7 (AAR24741.1), AtJAZ8 (ABG48454.1)AtJAZ9 (AAM10238.1), AtJAZ10 (NP001154713.1), AtJAZ11 (AAU15160.1), AtJAZ12 (AAK93690.1).

**Figure 2:**
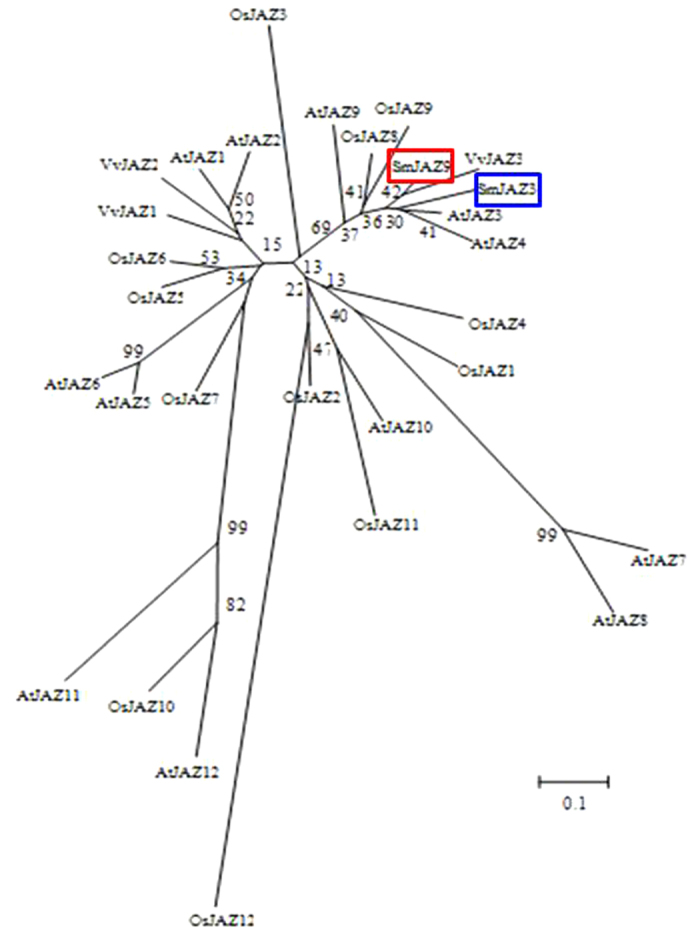
Phylogenic tree analysis of SmJAZs with other JAZs from *A. thaliana*, *Oryza sativa* and *Vitis vinifera* using Mega 5.1.

To isolate *S. miltiorrhiza* JAZ genes which are induced by MJ and functionally identify their roles in tanshinone biosynthesis, conserved domains of ZIM and Jas were used to blast *S. miltiorrhiza* transcriptome database. Two JAZ fragments were found, one lacked 3′ terminal (partial *JAZ3*) and the other contained complete 921 bp ORF (later named *JAZ*9). Based on sequences of JAZ domains, a primer F421 was designed to amplify 3′ terminal cDNA of the *JAZ3* together with the primer AUAP (Fig. S1) to get the complete coding sequence of *JAZ3*. The *SmJAZ3* gene described here encodes a 336-amino acid protein. Sequence alignment (Fig. 1) revealed that the two *JAZ*s share high amino acid sequence identity with JAZs from *Arabidopsis thaliana*. Phylogenetic analysis of selected JAZ proteins showed the one SmJAZ was highly homologous to AtJAZ3, thus we named it SmJAZ3 (Fig. 2). And the other SmJAZ contains a 921 bp ORF which encodes 306 amino acid residues (Fig. S1) showing the highest homology with AtJAZ9, accordingly, this *SmJAZ* was named as *SmJAZ9*. Both *SmJAZ3* and *SmJA*Z9 processed two conserved domains, the TIFY domain located near the N-terminus consists of 28 amino acids and the Jas motif in the C-terminal.

### Tissue expression patterns of *SmJAZ3* and *SmJAZ9*

**Figure 3:**
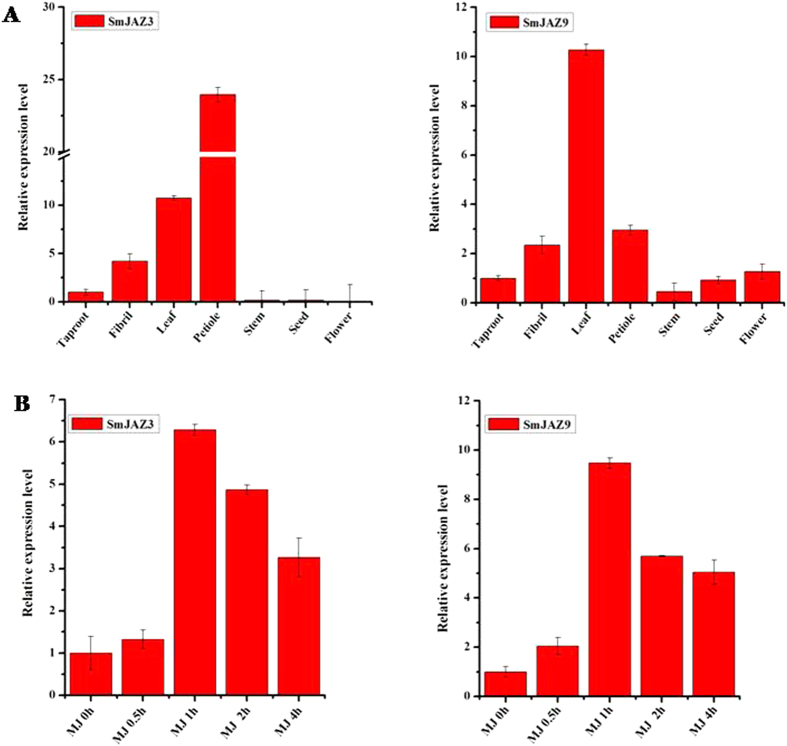
Expression profiles of *SmJAZ3/SmJAZ9* (**A**) Tissue expression patterns of *SmJAZ3/SmJAZ9*. Different tissues from two-year old *S. miltiorrhiza* were used for extraction of total RNA and analysis of expression profile. (**B**) Induction profiles of *SmJAZ3/SmJAZ9* after 100 μM MJ elicitation. Five time points were chosen at 0 h, 0.5 h, 1 h, 2 h and 4 h after MJ elicitation.

The expression profile of *SmJAZ3* and *SmJAZ9* in various *S.miltiorrhiza* tissues including taproot, fibril, leaf, petiole, stem, seed and flower was examined using qRT-PCR. High expression of *SmJAZ3* was detected in mature leaf and petiole, while low expression presented in taproot and fibril, and *SmJAZ3* expression was hardly observed in stem and reproductive organs, such as seed and flower (Fig. 3A). The expression of *SmJAZ9* was detected in almost all the tissues, highest *SmJAZ9* expression was found in mature leaf, less *SmJAZ9* expression was observed in petiole and fibril (Fig. 3A).

### Expression profiles of *SmJAZ3* and *SmJAZ9* induced by Methyl Jasmonate

*A. thaliana JAZs* have been reported to response to 100 μM methyl jasmonate at the earliest sampling time (0.5 hour). To investigate the induction effect of MJ on *SmJAZ3* and *SmJAZ9* expression, 2-month-old *S. miltiorrhiza* hairy roots were treated with 100 μM MJ and analyzed at five time points (0 h, 0.5 h, 1 h, 2 h, 4 h). The expression of *SmJAZ3* increased from 0.5 h and reached to the highest level at 1 h after MJ elicitation, which is nearly 6-fold higher than the control (Fig. 3B). This indicated that the *SmJAZ3* was responsive to MJ induction. The expression profile of *SmJAZ9* was similar to *SmJAZ3*, also increased to the highest level at 1 h after MJ treatment, which was 8 times higher than the control (Fig. 3B).

### Subcellular localization of *SmJAZ3* and *SmJAZ9*

**Figure 4:**
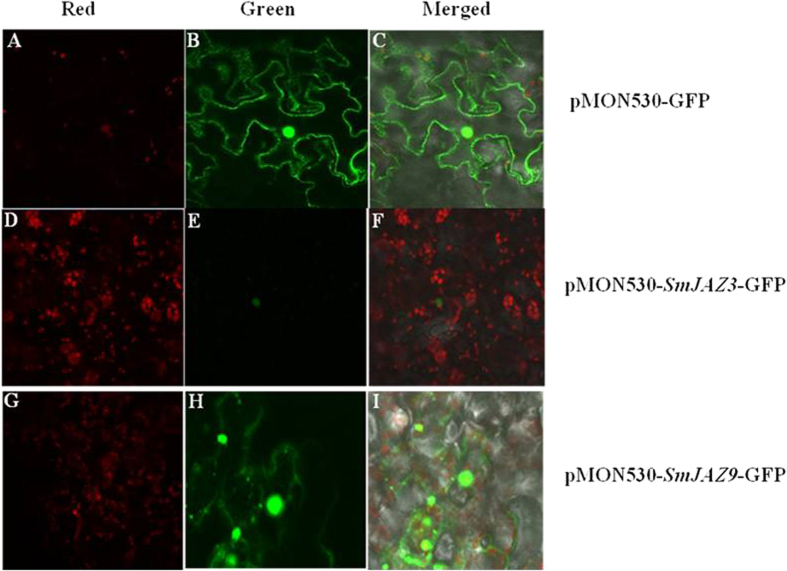
Transient expressions of the *SmJAZ3* and *SmJAZ9* in tobacco. The complete cDNA was inserted into the vector named *pMON530* with the restriction enzyme sites *Bgl*II and *Kpn*I. (**A–C**) the empty vector; (**D–F)**, transient expression of the *SmJAZ3* in tobacco; (**G–I**) transient expression of the *SmJAZ9* in tobacco.

To determine the Subcellular localization of *SmJAZ3* and *SmJAZ9*, the ORF of *SmJAZ3* and *SmJAZ9* were fused with the green fluorescent protein (GFP) in vector *pMON530*. Then the constructs were introduced into *Agrobacterium* strain ASE, respectively. Subcellular localization results showed that *SmJAZ3* was only localized to the nucleus while *SmJAZ9* was mainly in the nucleus but with some signal in the membrane in one-month-old tobacco (Fig. 4). This indicates that *SmJAZ3* and *SmJAZ9* are preferentially nuclear-localized, implying that they may function as transcriptional regulators.

### Generation of transgenic hairy root lines over-expressing or repressing *SmJAZ3/SmJAZ9*

Plasmid containing ORF of *SmJAZ3*/*SmJAZ9* or *anti-SmJAZ3*/*anti-SmJAZ9* under the control of the cauliflower mosaic virus (CaMV) 35 S promoter was transformed into *Agrobacterium* C58C1 and then transferred into *S. miltiorrhiza* to generate transgenic hairy root lines, respectively (Fig. S2). Totally, 38 *SmJAZ3* and 28 *SmJAZ9*-overexpressing hairy roots lines, as well as 36 *anti-SmJAZ3* and 36 *anti-SmJAZ9*-repressed hairy roots lines were generated.

### PCR analysis of genetically engineered hairy roots

Genomic DNA was isolated from all the hairy root lines individually and was used for PCR analysis using gene-specific primers to amplify the *CaMV35S* promoter and partial *SmJAZ3* or *SmJAZ9* gene. Plastid *pCAMBIA2300-SmJAZ3* and *pCAMBIA2300-SmJAZ9* were used as positive control. The blank vector *pCAMBIA2300*^+^-transformed lines and water were set as the negative controls. Representative results of PCR analysis of transgenic hairy roots were showed in Fig. S3. The PCR-positive rates of hairy root lines from *JAZ3*, *JAZ9*, *anti-SmJAZ3* and *anti-SmJAZ9* lines were 21/38 (55.3%), 14/28 (50.0%), 16/36 (44.4%) and 16/36 (44.4%) (Fig. S3). Our results suggested that *SmJAZ3* and *SmJAZ9* were successfully transformed into the genome of *S.miltiorrhiza*. Here we selected 5 *SmJAZ3*, 5 *SmJAZ9*, 3 *anti-SmJA*Z*3* and 3 *anti-SmJAZ9* of PCR-positive hairy root lines for further experiments.

### Effect on tanshinone biosynthetic genes by expression of *SmJAZ3* or *SmJAZ9* in transgenic hairy roots

**Figure 5:**
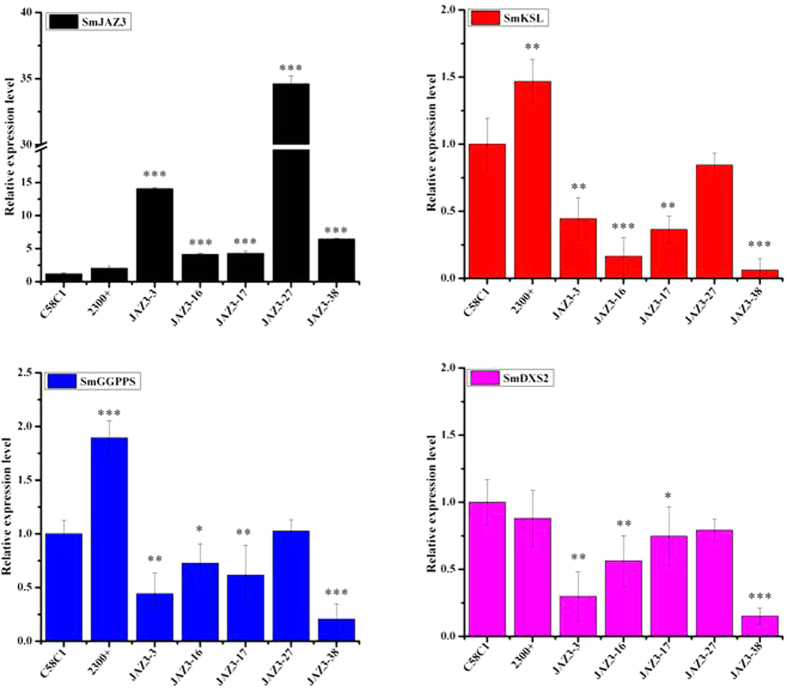
Expression levels of *SmJAZ3* and some tanshinone biosynthetic related genes in *S. miltiorrhiza* transgenic *SmJAZ3* hairy roots and non-transgenic hairy roots.

To determine whether the *SmJAZ3 or SmJAZ9* can affect tanshinone biosynthetic genes, expression profiles of several key tanshinone biosynthetic genes such as *SmGGPPS*, *SmDXS2* and *SmKSL* were examined, respectively. In the *SmJAZ3* overexpressing *S. miltiorrhiza* hairy root lines, *SmJAZ3* expression was up-regulated by 5–40 times in comparison to the control lines, while the transcripts of *SmGGPPS*, *SmDXS2* and *SmKSL* were all reduced with different level in the *SmJAZ3* overexpressing *S. miltiorrhiza* hairy root lines (Fig. 5). Among them, *SmKSL* showed the most significant decrease, nearly 20-fold lower in *SmJAZ3-38* line than the control (Fig. 5). Meanwhile, the key MEP pathway gene *SmDXS2* decreased to a lesser extent (Fig. 5). On the contrary, the expression of a few genes including *SmCPS* increased by 2–6 folds than the empty vector control, furthermore *SmCYP76AH1* did not show obvious change. In the *SmJAZ9*-overpressing hairy roots lines, the level of *SmJAZ9* were elevated by 5 times to the most, whereas the expression profiles of *SmGGPPS*, *SmDXS2* and *SmKSL* didn’t show similar expression pattern as those in *SmJAZ3*, indicated that *SmJAZ9* may have much weaker effect to repress biosynthesis of tanshinone when compared to *SmJAZ3*.

### Effect on tanshinone accumulation by expression of *SmJAZ3* or *SmJAZ9* in transgenic hairy roots

**Figure 6:**
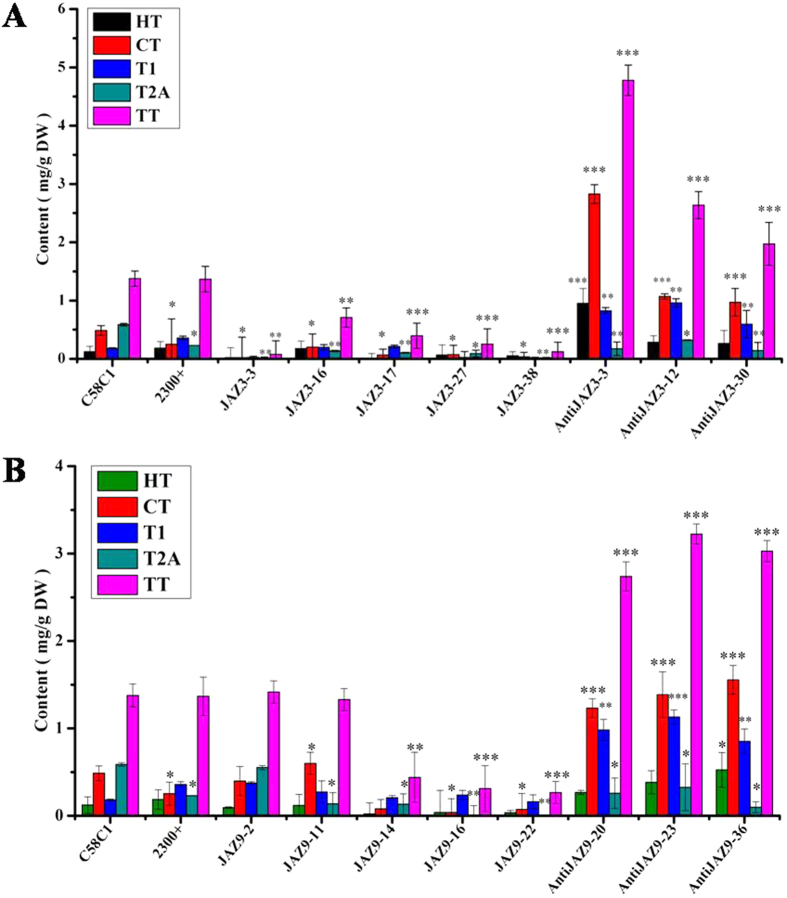
Analysis of tanshinone production from transgenic hairy root lines by HPLC. (**A**) Tanshinone content in *SmJAZ3* transgenic hairy root lines and *anti-SmJAZ3* transgenic hairy root lines, (**B**) Tanshinone content in *SmJAZ9* transgenic hairy root lines and *anti-SmJAZ9* transgenic hairy root lines. The control C58C1 hairy root cultures were generated from empty *Agrobacterium* C58C1 transformation. 2300^+^ hairy root cultures represents *pCAMBIA2300*^+^ -empty vector transgenic hairy roots. The values are means±S.D of triplicate analysis (P<0.05).

Tanshinone is synthesized from universal precursor IPP that derived through two different pathways in separate cellular compartments, the MVA pathway occurring in the cytosol and MEP pathway occurring in the plastids, while the MEP plays a more important role in tanshinone biosynthesis^23^. The contents of tanshinone including dihydrotanshinone (HT), cryptotanshinone (CT), tanshinone I (T1) and tanshinone IIA (T2A) in *S. miltiorrhiza* hairy root lines were determined by HPLC, and the results were shown in Fig. 6. Consistent with the gene expression profile, the accumulation of tanshinone in *SmJAZ3*-overexpressing lines appeared to decrease. Over-expression of *SmJAZ3* in hairy root produced evidently lower level of tanshinone ranging from 0.077 to 0.94 mg/g DW compared with the control (1.37 mg/g DW), tanshinone production in line *SmJAZ3-3* was as low as 0.077 mg/g DW, a 94% decrease. Transgenic hairy roots lines harboring *SmJAZ9* exhibited a slightly suppressive effect on the tanshinone biosynthesis, and the content of tanshinone decreased to 0.266 mg/g DW in *SmJAZ9-22* line. Whereas, the content of tanshinone increased to 4.78 mg/g DW in *anti*-*SmJAZ3-3* lines which was about 2.48-fold higher than the control, and it was about 3.22 mg/g in *anti-SmJAZ9-23* lines with 1.35-fold higher than the control. The above results inferred that the inhibitory effect of *SmJAZ3* on tanshinone biosynthesis is much stronger than *SmJAZ9*.

### SmJAZ9 protein interacts with AtMYC2

**Figure 7:**
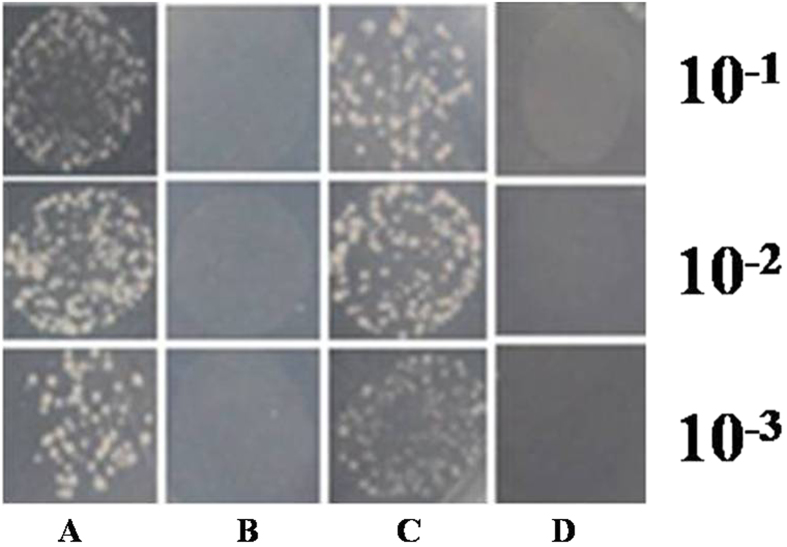
Yeast two-hybrid assay. pGBKT7-SmJAZ and pGADT7-AtMYC2 were co-transformed into Saccharomyces cerevisiae AH109 cells. Plasmids pGBKT7-AtJAZ9 and pGADT7-AtMYC2 were co-transformed into AH109 as the positivecontrol. (**A**) BD-SmJAZ9 +AD-AtMYC2 ; (**B**) BD-SmJAZ3+ADAtMYC2; (**C**) BD-AtJAZ9+AD-AtMYC2 (Positive control); (**D**) BD+AD (Negative control).

It has been reported that JAZ repressors interact with some transcription factors such as MYC2 which play an important role in JA-activated response. In order to test whether there is interaction between SmJAZs and AtMYC2 (MYC2-like proteins were not identified from *S. miltiorrhiza* so far), we fused the *SmJAZ3*, *SmJAZ9* to the *pGBKT7* vector (BD) and *AtMYC2* to the *pGADT7* vector (AD), followed by co-introduction of the above vectors into yeast cells *AH109* for Y2H. Plasmids *pGBKT7-AtJAZ9* and *pGADT7-AtMYC2* were co-transformed into *AH10*9 as the positive control and the empty vector *pGADT7* was co-transformed with *pGBKT7* constructs as a negative control (Fig. 7). Our results showed only SmJAZ9 can interact with AtMYC2 whereas SmJAZ3 couldn’t, which implied some degree of interaction of JAZ and MYC2 possibly existed in *S. miltiorrhiza* to regulate biosynthesis.

## Discussion

Being one kind of diterpenes with good pharmaceutical activities, tanshinone was essentially derived from two common precursors, isopentenl diphosphate (IPP) and dimethylallyl diphosphate (DMAPP)^29^,^34^,^35^. The two precursors were synthesized via two different pathways in separate cellular compartments, the MVA pathway occurring in the cytosol and MEP pathway in the plastids^23^. Metabolic engineering approach has been considered as an effective approach to increase the valuable metabolites in some medical plants^36^,^37^. Based on the successful isolation of several tanshinone biosynthetic genes such as *SmHMGR*, *SmDXR*, *SmDXS* and *SmGGPPS* from *S. miltiorrhiza*, the attempts to increase the tanshinone production in *S. miltiorrhiza* via metabolic engineering approach and combination with elicitor treatments have been reported^23^,^24^,^28^. However, due to lack of genome sequences for *S. miltiorrhiza*, several catalytic enzymes in the late steps of tanshinone synthesis still remain unknown. The rapid development of next-generation sequencing technology has greatly pushed the advance in transcription profile and new gene pools. Accumulations of tanshinone could be induced by various elicitors including plant hormone MJ, whereas little is known about molecular regulatory mechanism in tanshinone biosynthetic pathway. In this study, two JAZ genes *SmJAZ3* and *SmJAZ9* were cloned from *S. miltiorrhiza* and functionally identified as repressors involved in tanshinone biosynthesis. Bioinformatics analysis revealed that the two cloned *SmJAZ* genes showed high homology with *JAZ*s from *Arabidopsis* and contained typical ZIM and Jas domains, indicating those functional domains are conserved for plant JAZ family during evolution. The expression profiles under methyl jasmonate (MJ) elicitation revealed that expression of *SmJAZ3* and *SmJAZ9* was quickly MJ-responsive, which suggested that *SmJAZ3* and *SmJAZ9* may be involved in MJ signal pathway and its related activated responses, in good agreement with those *JAZ*s previously reported in model plant *A. thaliana*^17^. It has been reported that methyl jasmonate could promote SCF^COI1^ and JAZ to generate protein complex and then 26 S proteomes were recruited to degrade JAZ proteins to liberate positive regulators such as MYC2 whose transcriptional activation was repressed by JAZs under normal condition^5^,^7^,^17^. It may be hypothesized that MJ may promote SCF^COI1^-like protein interact with JAZs and make JAZ proteins degraded by 26 S proteomes in *S. miltiorrhiza*, and then some positive transcription regulators such as MYC2 were released from repression by JAZs to transcriptionally activate the expression of some tanshinone biosynthetic genes and correspondingly modulate tanshinone biosynthesis. SmJAZ9 could interact with AtMYC2, implied that the above hypothesis may exist in *S. miltiorrhiza*, which needed to be investigated further in the future. Over-expression of *SmJAZ3* and *SmJAZ9* in *S. miltiorrhiza* hairy roots resulted in reduced accumulation of tanshinone while down-regulation of *SmJAZ3* and *SmJAZ9* effectively improved the production of tanshiones. These indicated that *SmJAZ3* and *SmJAZ9* play important roles in tanshinone biosynthesis and act as repressive components in the JA signal pathway for tanshinone production, which paves a way to dissect molecular mechanism in details in the future.

JAZ proteins belong to a larger group of ZIM-domain proteins, named after the putative transcription factor Zinc-finger inflorescence meristem (ZIM). ZIM family members were assigned to this group according to a conserved 28 amino acid ZIM motif. In addition to ZIM, JAZ family members also have the highly conserved Jas motif of 26 amino located near the C-terminus, it is now well established that Jas motif participates in protein-protein interaction with both transcription factors like the member of bHLH family (MYC2) and COI1. In the present study, it was found that SmJAZ9 protein can interact with the AtMYC2 by yeast two hybrids (Y2H), which was consistent with former reports that the JAZ family has the character to interact with MYC2^38^,^39^. We didn’t detect SmJAZ3 protein’s interaction with the AtMYC2 by Y2H, which may be attributed to genes from different plant species with uncertain complexity. The above data suggested that there may be a potential MYC2-like transcription factor in *S. miltiorrhiza*, which may be the target of JAZ proteins and play an important role in accumulation of tanshinone as positive regulator. Whether the SmJAZs have an interaction with the potential SmMYC2 needed to be further investigated. Thus work towards isolation and identification of MYC2-like transcription factor from *S. miltiorrhiza* may further decipher this action mechanism.

In *A. thaliana* and some other model plants, several reports have revealed the direct involvement of JA and JAZ proteins in growth and development processes such as secondary growth^40^, phytochrome, anthocyanin accumulation, trichrome initiation^41^, stamen development^42^, and defense response against biotic or abiotic stresses^43^,^44^,^45^. Our work revealed that over-expression of *SmJAZ3* in *S. miltiorrhiza* hairy roots down-regulated several tanshinone biosynthesis key genes such as *SmDXS2*, *SmK*SL and *SmGGPPS* in comparison to the control lines. At the same time, the production of tanshinone was decreased to 0.077 mg/g DW in *JAZ3* over-expressing line *JAZ3-3* in comparison to the control line (1.37 mg/g DW). While down regulated expression of *JAZ3* in *anti-JAZ3-3* line increased the content of tanshinone to 4.78 mg/g DW, which was 2.48-fold higher than the control. In transgenic *SmJAZ9* line, average production of tanshinone was 1.32 mg/g DW, whereas, the content of tanshinone increased to 3.22 mg/g DW in anti-*JAZ9-23* lines, a 1.35-fold increase as the control. These results reflect that the *SmJAZ3* has much stronger suppressive effect on tanshinone biosynthesis than the *SmJAZ9*. Furthermore it was found that *SmJAZ3* and *SmJAZ9* acted as repressors in tanshinone biosynthesis. The above data testified the role of JA in regulating tanshinone biosynthesis genes and thereby affecting the production of tanshinone in *S. miltiorrhiza*. However, further investigations are needed to figure out whether SmJAZs interact with SmMYC2 or other transcription factors, how and which SmJAZ they interact with. We also need to further elucidate how the JA signaling functions or whether it cross talks with other signaling pathways in *S. miltiorrhiza*. Overall, this study extends our understanding in the molecular regulation of tanshinone biosynthesis induced by MJ signal. Our work indicated JAZs such as *SmJAZ3* and *SmJAZ9* play important role in regulation of tanshinone biosynthesis, it may be involved in other secondary metabolism. The isolation and functional characterization of the two JAZs from *S. miltiorrhiza* paves a way to further dissect molecular mechanism and manipulate tanshinone production in plant.

## Additional Information

**How to cite this article**: Shi, M. *et al.* Methyl jasmonate induction of tanshinone biosynthesis in *Salvia miltiorrhiza* hairy roots is mediated by JASMONATE ZIM-DOMAIN repressor proteins. *Sci. Rep.* **6**, 20919; doi: 10.1038/srep20919 (2016).

## Supplementary Material

Supplementary Information
