# Supplementary material for: Methyl jasmonate induction of tanshinone biosynthesis in Salvia miltiorrhiza hairy roots is mediated by JASMONATE ZIM-DOMAIN repressor proteins
Source: Sci Rep. 2016 Feb 15;6:20919. doi: 10.1038/srep20919 (PMC4753458; doi:10.1038/srep20919)
Supplement: Supplementary Information [file srep20919-s1.pdf]

Methyl jasmonate induction of tanshinone biosynthesis in *Salvia miltiorrhiza* hairy roots is mediated by JASMONATE ZIM-DOMAIN repressor proteins

Min Shi[1,2*], Wei Zhou[2*], Jianlin Zhang[2], Shengxiong Huang[3], Huizhong Wang[1], Guoyin Kai[2]

[1]Zhejiang Provincial Key Laboratory for Genetic Improvement and Quality Control of Medicinal Plants, Hangzhou Normal University, Hangzhou 310018, People's Republic of China; [2]Institute of Plant Biotechnology, Development Center of Plant Germplasm Resources, College of Life and Environment Sciences, Shanghai Normal University, Shanghai 200234, People's Republic of China; [3]Kunming Institute of Botany, Chinese Academy of Sciences, Kunming 650201, People's Republic of China.

*These authors contributed equally to this work

Correspondence and requests for materials should be addressed to G.Y.K.

(email: gykai@hotmail.com, guoyinkai@yahoo.com).

```
1     atggagagagatttcatggggttgaaccctaaggatgctgctgttaaggaagagcttgtc
       M  E  R  D  F  M  G  L  N  P  K  D  A  A  V  K  E  E  L  V
61    gagggcggctgcgaagaatctggatttgcaaggagctctagcgttccatggcctacaaac
       E  G  G  C  E  E  S  G  F  A  R  S  S  S  V  P  W  P  T  N
121   aaggtgtctggcctccctcaatttatgtcttcgaggtgcaaacaagatgagaaggcaatg
       K  V  S  G  L  P  Q  F  M  S  S  R  C  K  Q  D  E  K  A  M
181   aagaatgggcagctgggatatcatatgcatccactttccatgaagccacaattccttggt
       K  N  G  Q  L  G  Y  H  M  H  P  L  S  M  K  P  Q  F  L  G
241   ggaagtactcatacagctccgcatcatagtcttccttcagctgactctacggcaggaata
       G  S  T  H  T  A  P  H  H  S  L  P  S  A  D  S  T  A  G  I
301   actgaacaatggatcaattctaaggcttctaatgctcctgctcagttgactatttttat
       T  E  Q  W  I  N  S  K  A  S  N  A  P  A  Q  L  T  I  F  Y
361   ggcggaacagtgtgtgtctttgatgacatttcccctgagaaggctcaagcgatcatgctt
       G  G  T  V  C  V  F  D  D  I  S  P  E  K  A  Q  A  I  M  L
421   ttggctggaaatgtgtatgttcaatctatgatgactcaatcgaaagttcacatgcaagca
       L  A  G  N  V  Y  V  Q  S  M  M  T  Q  S  K  V  H  M  Q  A
481   cctgcctccaaagttccagcagcagatcaaccattggctaaccaatccatgaatactcca
       P  A  S  K  V  P  A  A  D  Q  P  L  A  N  Q  S  M  N  T  P
541   ccaagttctggactcccaagccctatgtctgtttcttctcacccaattgatcagtccagt
       P  S  S  G  L  P  S  P  M  S  V  S  S  H  P  I  D  Q  S  S
601   gttccagggactaacaataatgacatcaagttatctaagatcgctggcatgtcaaccgcc
       V  P  G  T  N  N  N  D  I  K  L  S  K  I  A  G  M  S  T  A
661   cttgttaacaatactgaacctcctagagtgatgtcatctgttgctgcatctgctttaatg
       L  V  N  N  T  E  P  P  R  V  M  S  S  V  A  A  S  A  L  M
721   tcatcagctgttccacaggcacggaaagcatctttggctcgatttttggagaagcgtaag
       S  S  A  V  P  Q  A  R  K  A  S  L  A  R  F  L  E  K  R  K
781   gagagggtgatgagtgcagcagcaccatacaaccagggcaagaaggcagctgattgtaaa
       E  R  V  M  S  A  A  A  P  Y  N  Q  G  K  K  A  A  D  C  K
841   acaccagagtcgaatgatttggattctcggctacatctgggaccttctctagttccgtc
       T  P  E  S  N  D  F  G  F  S  A  T  S  G  T  F  S  S  S  V
901   tccgtcagcaaggatgactga
       S  V  S  K  D  D  *
```

```
1     atggagagagatttcatgggtttgtctgtgaagcaggaggttcctgatgaaataattgat
       M  E  R  D  F  M  G  L  S  V  K  Q  E  V  P  D  E  I  I  D
61    gctgcttcagtgagaagcttaccaatgcagtggtcattctccaacaagggctctgctctt
       A  A  S  V  R  S  L  P  M  Q  W  S  F  S  N  K  G  S  A  L
121   cctcagctcttgtcttttcaaggtgctcaagaagacaaacaacccaaaactggttttaat
       P  Q  L  L  S  F  Q  G  A  Q  E  D  K  Q  P  K  T  G  F  N
181   tctcttgcatcatctggattggttaccttaactaccgatgttttcgactccgatcacagc
       S  L  A  S  S  G  L  V  T  L  T  T  D  V  F  D  S  D  H  S
241   cagtttcctgcttcacagaaaagccatgttcctgagaagcaaggtggggttcgttacacg
       Q  F  P  A  S  Q  K  S  H  V  P  E  K  Q  G  G  V  R  Y  T
301   gtgacaacatacggagacacacacaatatacgtcgtgcaatcaccacttcaccagcctcc
       V  T  T  Y  G  D  T  H  N  I  R  R  A  I  T  T  S  P  A  S
361   tacgctgctcctgtggccggatttccagtggcgaatcctctacaagccaagccttctaac
       Y  A  A  P  V  A  G  F  P  V  A  N  P  L  Q  A  K  P  S  N
421   agcgctgccgttggaaccactgatttaggaactcgtgcaagatctcgaacacgcctgct
       S  A  A  V  G  T  T  D  F  R  N  S  C  K  I  S  N  T  P  A
481   cagctaaccatcttctacaacggctcagtatgcgtgtacgacgacatttctcccgagaag
       Q  L  T  I  F  Y  N  G  S  V  C  V  Y  D  D  I  S  P  E  K
541   gctcaagctattatgctattggctggaaatgcacattcagtggctcctaaggcgacgcct
       A  Q  A  I  M  L  L  A  G  N  A  H  S  V  A  P  K  A  T  P
601   cctgcagttcccgtccaggccgccatgcctagatgttcggttcctgacaggctaaccata
       P  A  V  P  V  Q  A  A  M  P  R  C  S  V  P  D  R  L  T  I
661   aaccagtcttatgcaaccacacctcgtcgttccagccctgtccctatgacccccattagc
       N  Q  S  Y  A  T  T  P  R  R  S  S  P  V  P  M  T  P  I  S
721   gtctcccagtctgctaataagccatctggagctcgtatgtcgcctcctatcaatgctgag
       V  S  Q  S  A  N  K  P  S  G  A  R  M  S  P  P  I  N  A  E
781   cctctgaaaacagtcattcctctaggatctgcaagctttctatcatcagacactgtgcct
       P  L  K  T  V  I  P  L  G  S  A  S  F  L  S  S  D  T  V  P
841   cagtttcgcaaaaaatctctagcccggttcttggagaaacgcaaggagagggtgatcagt
       Q  F  R  K  K  S  L  A  R  F  L  E  K  R  K  E  R  V  I  S
901   gcttcaccttatggtgagtgtcaatctggggattatagcgctgccggagcaggcgcggtg
       A  S  P  Y  G  E  C  Q  S  G  D  Y  S  A  A  G  A  G  A  V
961   agcttgtcgatgagctcttcaggatcttgtccagttccagctgccaattga
       S  L  S  M  S  S  S  G  S  C  P  V  P  A  A  N  *
```

Figure S1 The coding sequences of *SmJAZ3* and *SmJAZ9*.

SmJAZ3 gene contains a 1011 bp ORF which encodes 336 amino acid residues and *SmJAZ9* contains a 921 bp ORF which encodes 306 amino acid residues.

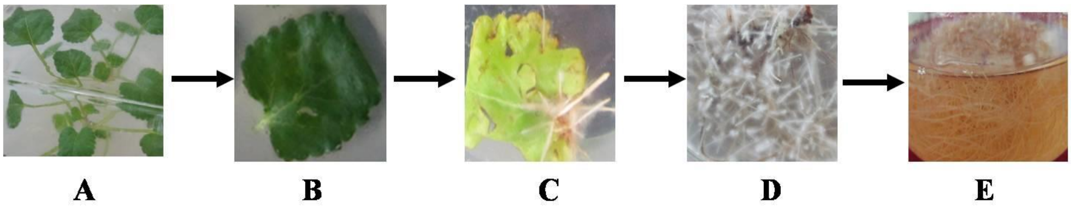

Figure S2 Procedures of generation of transgenic *S.miltiorrhiza* hairy roots.

The sterile plant of *S. miltiorrhiza* plants. (B) Pre-cultivation and co-cultivation the leaves of *S. miltiorrhiza*. (C) Hairy roots after excising from the leaves. (D) The single lines of transgenic hairy roots. (E) The hairy roots in the flask.

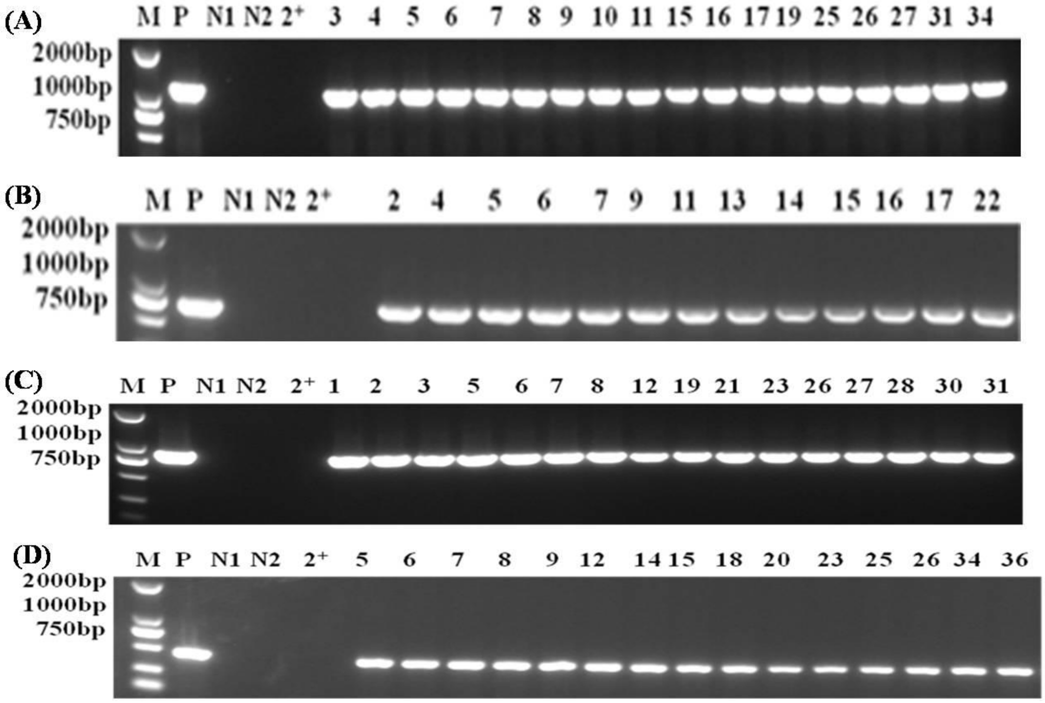

Figure S3 PCR analysis of transgenic hairy root lines.

Representative PCR analysis for the presences of *SmJAZ3* (A) and *SmJAZ9* (B); Inhibition of *SmJAZ3* (C) and *SmJAZ9* (D) in transgenic lines, respectively. M DL-2000 Marker, P positive control (vector contains corresponding gene), N1 negative control 1 (wild *S. miltiorrhiza* DNA), N2 negative control 2 (water), 2^+ hairy root generated from blank vector transformation (*pCAMBIA2300*$^+$)
